# Embedding-based Silhouette community detection

**DOI:** 10.1007/s10994-020-05882-8

**Published:** 2020-07-27

**Authors:** Blaž Škrlj, Jan Kralj, Nada Lavrač

**Affiliations:** 1 Jožef Stefan Institute, Jamova 39, 1000 Ljubljana, Slovenia; 2 Jožef Stefan International Postgraduate School, Jamova 39, 1000 Ljubljana, Slovenia; 3 CosyLab, Gerbičeva ulica 64, 1000 Ljubljana, Slovenia; 4 University of Nova Gorica, Vipavska 13, 5000 Nova Gorica, Slovenia

**Keywords:** Node embedding, Community detection, Network analysis, Unsupervised learning

## Abstract

Mining complex data in the form of networks is of increasing interest in many scientific disciplines. Network communities correspond to densely connected subnetworks, and often represent key functional parts of real-world systems. This paper proposes the embedding-based Silhouette community detection (SCD), an approach for detecting communities, based on clustering of network node embeddings, i.e. real valued representations of nodes derived from their neighborhoods. We investigate the performance of the proposed SCD approach on 234 synthetic networks, as well as on a real-life social network. Even though SCD is not based on any form of modularity optimization, it performs comparably or better than state-of-the-art community detection algorithms, such as the InfoMap and Louvain. Further, we demonstrate that SCD’s outputs can be used along with domain ontologies in semantic subgroup discovery, yielding human-understandable explanations of communities detected in a real-life protein interaction network. Being embedding-based, SCD is widely applicable and can be tested out-of-the-box as part of many existing network learning and exploration pipelines.

## Introduction

Mining complex data in the form of networks is of increasing interest in many scientific disciplines: social, biological, manufacturing and similar systems can be represented and analyzed using network-based approaches. Recently developed embeddings technology offers advancements in representation learning (Zhang et al. 2018) from different data formats, including learning representations of network data, such as network node embeddings (Cai et al. 2018). Even though such embeddings are commonly used for supervised learning, such as node classification and link prediction, less attention is devoted to the study of how the latent *organization* of a network can be automatically extracted from node embeddings in an *unsupervised* manner.

Real-world complex networks are commonly investigated in terms of their meso-scale topological structure, such as communities (Harenberg et al. 2014). Algorithms such as InfoMap (Rosvall et al. 2009a; Schaub et al. 2017) and Louvain algorithm (De Meo et al. 2011) are well established for the task of *community detection*. Identification of communities for example offers insights into the inner workings of cellular function, mobile and transportation networks, and helps with the identification of potential security threats.

**Fig. 1:**
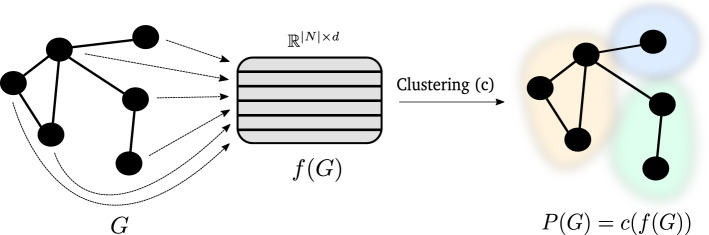
Schematic representation of the proposed embedding-based SCD approach. The input network *G* (comprised of a set of nodes *N*) first gets mapped to a latent *d*-dimensional vector space via *f*. The vectors are clustered (via *c*) to obtain the final partition *P*(*G*)

In this work we explore whether the state-of-the-art machine learning techniques for representation learning can be used effectively in unsupervised network analysis. The goal is to bridge the two domains, network embedding and community detection, by demonstrating that node embeddings—when clustered—offer insights into network community structure. Being able to detect communities from embeddings directly could greatly reduce the complexity of existing computational pipelines, comprised of multiple different methods, as well as speed up the development process. The purpose of this work is thus to explore whether node embeddings can be used to build an explainable and scalable community detection algorithm. This is achieved by applying a geometric measure of partition quality (the Silhouette score) on node embeddings directly. The proposed approach is summarized in Fig. 1.

The contributions of this work can be summarized as follows:

1. We show how the community detection problem can be cast as a network embedding clustering task via optimization of the Silhouette score.
2. The method is evaluated against strong, established baselines on a large synthetic network collection comprised of 234 networks, where it offers competitive performance.
3. It is also evaluated on a real-world network, comprised of E-mail communities, where it outperforms the state-of-the-art.
4. The proposed method scales to large networks and can be used with arbitrary node representation learners, which was demonstrated using SCD with two distinct node embedding methods.
5. We reimplemented NetMF (one of the embedding methods used) in PyTorch (Paszke et al. 2017) for maximum efficiency and for improved scalability. The method runs on GPUs, as well as in parallel on CPUs. The re-implemented node embedding methods, NetMF, and the PPRS algorithm, are now publicly available for non-commercial use.
6. On a real-life biological network we showcase that the communities detected with SCD can be *interpreted* via external background knowledge in the form of ontologies, yielding simple rules that describe (in a human-understandable way) the detected communities.

In summary, the conceptual novelties of this paper are further outlined below. First, the Silhouette score was extensively analysed, which led us to a novel theoretical contribution that helped us reduce the search space of the numbers of communities. Second, extensive empirical evidence on a set of synthetic and real networks indicates that the proposed method performs similarly well to state-of-the-art Louvain or InfoMap algorithms, yet operates differently and is more general, as node embeddings can be used also for other down-stream tasks. Third, as node embedding-based models are not explainable, we show how inductive rules can be inferred from the communities by exploiting the domain background knowledge, demonstrating a potentially interesting link to human-understandable descriptions of the obtained communities with domain background knowledge; this connection was not yet demonstrated in the considered embedding-based setting.

The rest of this paper starts with the related work in Sect. 2. Section 3 presents the proposed embedding-based SCD methodology, which is the main contribution of this paper. The experimental setting and the quantitative evaluation of community detection capabilities of SCD are presented in Sect. 4. Section 5 presents the methodology and the results of qualitative interpretation of discovered communities on a selected use case. We summarize the obtained results and present the plans for further work in Sect. 6.

## Related work

The published work, related to advances presented in this paper, can be split into three major groups, presented in this section.

First, the Sect. 3 introduces a new method, the performance of which is tested in Sect. 4—this method is based on ideas from network embedding and community detection, both topics of network analysis (Sect. 2.1). In Sect. 5, the proposed method is used in analysis of a biological data set using methods of subgroup discovery (presented here in Sect. 2.2) and, more specifically, using the approach community-based semantic subgroup discovery (CBSSD), presented here in Sect. 2.3.

### Network analysis

This section starts by presenting the state-of-the-art methods for network embedding in Sect. 2.1.1, followed by the presentation of selected community detection methods in Sect. 2.1.2.

#### Network embedding

A series of the recently introduced methods from the field of representation learning attempt to learn from complex networks by first transforming them into a vectorized form, and then performing a desired down-stream learning task. Such *embeddings* (real-valued, vector representations of nodes) are for example useful for construction of large-scale recommender systems and similar tasks (Zhao et al. 2016). Here, for example, individuals can be recommended sets of items based on their embeddings’ similarity with other individuals.

Some of the well known node embedding algorithms include DeepWalk (Perozzi et al. 2014), its extension node2vec (Grover and Leskovec 2016), LINE (Tang et al. 2015), struc2vec (Ribeiro et al. 2017) and PTE (Tang et al. 2015). Many of these approaches were inspired by word2vec (Mikolov et al. 2013), the approach initially targeted for learning distributed representations of words. The graph-based approaches leverage similar idea and represent nodes as words and sample their neighborhoods to determine their representations in a latent space. The common property of these algorithms is that they perform the so-called “shallow embedding”, exploiting only a given network’s adjacency structure. On the other hand, apart from the adjacency structure, methodology from the growing field of geometric deep learning attempts to also exploit various node or edge *attributes*, present in real-world networks. For example, instead of only considering protein-protein interactions, this branch of methods can also consider e.g., a given protein’s molecular properties that are encoded as separate attributes for each node.

Intensive development of such methods started with the recently introduced graph convolutional neural networks (Defferrard et al. 2016; Kipf and Welling 2017), as well as for example the graph attention networks (Veličković et al. 2017), graphSAGE (Hamilton et al. 2017) and many others, recently summarized in Cai et al. (2018), Wu et al. (2019).

The common point to all of the aforementioned methods is, they mostly produce real-valued matrices, representing various aspects of a network, let that be the embeddings of desired subnetworks or just network nodes. Such vectorized form of e.g., nodes is a suitable input for the body of well established unsupervised pattern detection methods, such as for example the k-means (Bachem et al. 2016) or K-medoids families (Park and Jun 2009) of algorithms. Such clustering methodology has been in widespread use in the statistics and machine learning communities since the 1960s. Cluster quality can be evaluated with several measures, including Davies-Bouldin Index (Davies and Bouldin 1979), Calinski-Harabasz Index (Kozak 2012), Fowlkes-Mallows scores (Fowlkes and Mallows 1983) and the Silhouette score (Rousseeuw 1987). The latter, used in this work, is described in detail in Sect. 3.3.1.

#### Community detection

The field of community detection attempts to identify densely connected subnetworks with relevant, potentially causal meaning. As searching the space of all communities is prohibitively expensive (Brandes et al. 2006), heuristic-based approaches are developed, sourcing their principles from many fields of science, including physics, biology, sociology and other (Harenberg et al. 2014; Lancichinetti and Fortunato 2009; Honghao et al. 2013).

Established community detection algorithms operate on a network’s adjacency structure directly, and are thus specialized only for this task. Examples of such algorithms include the Louvain algorithm (De Meo et al. 2011), which maximizes the *modularity score* that approximates the network’s connectivity patterns so that densely connected parts of the network remain grouped. Another well established algorithm is InfoMap (Rosvall et al. 2009a), which operates using the ideas from the information theory. It encodes sampled random walks and attempts to find codewords, i.e. structures representing communities, so that their length is minimized. Intuitively, it samples the “information flow” across the network and maximizes such network partition, so that the flow remains captured in densely connected parts of a given network. Both InfoMap and Louvain algorithms scale to massive, real-world networks. The InfoMap algorithm offers also the insight into hierarchical community organization, which is commonly present in real-world networks. For example, social networks can be observed at the level of small groups, as well as e.g., whole organizations. Other recently introduced algorithms—that perform approximately the same as InfoMap and Louvain algorithms—include, for example, Grothendieck’s inequality communities (Guédon and Vershynin 2016) and SCORE (Jin 2015). We refer the reader to (Harenberg et al. 2014) for a more extensive overview.

Quality of community detection can be evaluated with several measures, including ARI (Adjusted Rand Index) (Rand 1971), NMI (Normalized Mutual Information) (Thomas and Cover 1991; Toni et al. 2009), as well as Modularity Score (Clauset et al. 2004). These measures, used also in this work, are presented in Sect. 4.1.5.

### Subgroup discovery

This section explains the subgroup discovery methodology in Sect. 2.2.1, followed by presenting the related work in semantic subgroup methodology in Sect. 2.2.2.

#### Background

Subgroup discovery is a field of research tackling the task of *rule learning*, a machine learning task, where given a set of target classes and a set of instances, the goal is to identify significant patterns in the data. The best rules are found by optimizing a scoring function, that can, for example, account for rule uniqueness and (predictive) performance. Rules represent human-understandable symbolic descriptions of a given data set, and are as such useful where a given method’s explainability matters.

In this work, we focus on subgroup discovery (SD), a subfield of supervised descriptive rule induction (Novak et al. 2009). Subgroup discovery is a subfield of rule learning focused on discovering rules from *class-labeled* data. In particular, subgroup discovery aims to find subsets of labelled data that share similar characteristics (i.e., have the same *explanation*) and are also labelled by the same class (i.e., share a *TargetClass*). In short, SD searches for rules of the form TargetClass $\leftarrow$ Explanation. These rules are traditionally learned via coverage-based approaches (Fürnkranz et al. 2012).

Note that the goal of subgroup discovery, as defined above, is similar to that of classification, with an important difference. The emphasis in SD is on inducing individual explainable patterns, whereas classification emphasizes the construction of complete models which are not necessarily explainable. Unlike in classification, the set of explainable rules, discovered by SD algorithms, is itself the goal of SD tasks. This contrasts with classification tasks, where such rules would present a means to an end—achieving good classification performance on unseen data.

In this work, we used a specialized subgroup discovery algorithm Hedwig (Adhikari et al. 2016; Vavpetič et al. 2013; Vavpetič 2017). This algorithm addresses a particular subfield of Subgroup discovery named Semantic Subgroup Discovery, explained in more detail in the following section.

#### Semantic subgroup discovery

Semantic subgroup discovery (SSD) (Langohr et al. 2012; Vavpetič et al. 2013) is a field of subgroup discovery, which uses ontologies as background knowledge in the subgroup discovery process. This methodology is capable of inducing rules from classification data, where class labels denote the groups for which descriptive rules are learned. In SSD, ontologies are used to guide the rule learning process. For example, the Hedwig algorithm (used in this work) accepts as input a set of class-labeled training instances, one or several domain ontologies, and the mappings of instances to the relevant ontology terms. Rule learning is guided by the hierarchical relations between the considered ontology terms. Hedwig is capable of using an arbitrary ontology to identify latent relations explaining the discovered subgroups of instances. Just like in regular subgroup discovery, Hedwig results in descriptions of target class instances as a set of rules of the form TargetClass $\leftarrow$ Explanation. The contribution of Hedwig is that the condition (the explanation) is a logical conjunction of terms from the domain ontologies mapped to the data. Hedwig was successfully applied in the biomedical domain (Adhikari et al. 2016; Škrlj et al. 2019a), supports RDF-encoded inputs, and is suitable for working with collections of background knowledge ontologies. Rule learning performed by Hedwig is guided by the hierarchical relations between the considered ontology terms.

Another novelty of the Hedwig algorithm is the method of learning the rules. As explained in Sect. 2.2.1, class-labeled rules are usually learned via coverage-based approaches. In contrast, Hedwig uses a specialized *beam search* procedure to discover the rules. Hedwig keeps *b* rules in its search beam ($|Beam| = b$), improving and refining them while traversing the search space of all possible rules. After concluding its search, the *b* rules, remaining on the beam, are the output of the algorithm.

For an interested reader we here explain the formulation for rule induction used by the Hedwig algorithm. The presented formulation consists of two objectives, rule uniqueness and rule quality, which together form the joint scoring function as follows:

1 $$\begin{aligned} {\mathfrak {R}}_{opt} = \mathop {\hbox {arg max}}\limits _{{\mathfrak {R}}}\frac{\sum _{R \in {\mathfrak {R}}}\epsilon (R)}{\sum _{\begin{array}{c} R_{i},R_{j}\in {\mathfrak {R}} \end{array}}|Cov(R_{i}) \cap Cov(R_{j})|+1};\quad i \ne j \end{aligned}$$

where ${\mathfrak {R}}$ represents a candidate set of rules, $R \in {\mathfrak {R}}$ represents a single rule, and $Cov(R_i)$ denotes the set of examples covered by $R_i$.

Hedwig aims to maximize the numerator of Eq. 1 in order to maximize rule quality of a set of rules. At the same time, it searches for rules that cover different parts of the example space, which is achieved by minimizing the denominator, i.e. minimizing the intersection of instances covered by different rules $R_{i}$ and $R_{j}$. In Hedwig, a set of rules (a beam of size *b*) is iteratively refined during the learning phase using a selected refinement heuristic, such as for example lift or weighted relative accuracy. The algorithm yields multiple different rules that represent different subgroups of the data set being learned on.

### Community-based semantic subgroup discovery

**Fig. 2:**
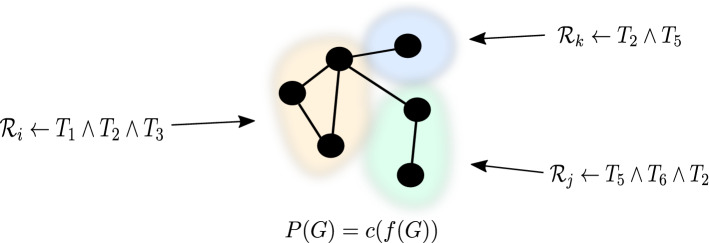
In community-based semantic subgroup discovery, individual rules, such as ${\mathcal {R}}_{i}$ ${\mathcal {R}}_{j}$ and ${\mathcal {R}}_{k}$, represent human-understandable descriptions, comprised of terms $T_i$ of individual communities

Community-based semantic subgroup discovery (CBSSD) is an approach that unifies community detection (presented in Sect. 2.1.2) and semantic subgroup discovery (presented in Sect. 2.2.2). The CBSSD step, illustrated in Fig. 2, can be understood as a post-hoc analysis to the embedding-based Silhouette Community Detection (SCD) algorithm proposed in Sect. 3. Note that this community explanation step is part of qualitative analysis, as the obtained patterns do not necessarily represent causal mechanisms—this part is commonly discussed with domain experts based on additional experimental evidence.

Following (Škrlj et al. 2018, 2019a), we next discuss how the semantic subgroup discovery algorithm Hedwig was adapted for the task of Community-based Semantic Subgroup Discovery (CBSSD), i.e., for *community enrichment* by finding sets of rules which uniquely describe a given community. We next describe some of the key ideas of CBSSD, yet direct an interested reader to Škrlj et al. (2019a) for extensive technical details and computational complexity analysis.

Let $P_1 \dots P_n$ represent individual communities returned by a community detection algorithm, such as SCD. Given background knowledge in the form of an ontology ${\mathcal {B}}$ and an injective mapping from a graph’s nodes to such knowledge $m:N \rightarrow {\mathcal {B}}$, Hedwig is used as follows: Each Partition is considered as a target class, whilst the remainder of the network is compared against. Thus, for *i*-th partition, rules of the form $P_i \leftarrow T_1 \wedge T_2 \wedge \dots \wedge T_n$ are learned, where $T_1$, $T_2 \dots T_n$ are elements of ${\mathcal {B}}$. For each community, a set of rules is learned, where the number of rules depends on the beam size used (input parameter).

As the first step of the CBSSD methodology consists of community detection, a task performed by the SCD algorithm, the two algorithms are intrinsically complementary, leading us to test a framework employing both of them in an exploratory setting, described in Sect. 5.

## Silhouette community detection

In this section, we present the proposed embedding-based method named Silhouette community detection (SCD). We begin by describing the general setting and the rationale that led us to the proposed approach. We continue by describing the notions of network embedding, as well as the Silhouette score we adapt for the task of community evaluation. We finally present the formal description of the proposed approach along with the analysis of its computational and spatial complexity.

### Definitions

This section serves as the introduction to the concepts used throughout this work. We first define the types of networks we consider, followed by definition of the community detection task.

#### Definition 1

(Weighted network) Formally, a weighted network *G* is a tuple, (*N*, *E*, *w*), where *N* is the set of nodes and *E* is the set of edges defined as unordered node pairs. The weight function *w* maps from edges to the field of real numbers, i.e. $w: E \rightarrow {\mathbb {R}}^+$, assigning a weight to each edge.

The proposed method also naturally handles unweighted networks, which can be understood as weighted networks where all weights are set to 1.

#### Definition 2

(Network node embedding) Given a network $G=(N, E, w)$, a network node embedding of *G* is a mapping $f: N \rightarrow {\mathbb {R}}^{d}$, where *N* is the set of network nodes. The value *d* is a parameter of the embedding referred to as the latent dimension of the output vector space.

We do not explicitly define the properties of *f*, as the wealth of existing methods exploits various aspects of *G*. Note that when the value of the embedding dimension *d* is 2, the embedding is a collection of pairs of real values, which can easily be visualized. The goal of network embedding methods is to maintain the relevant graph-topological properties in the obtained vector space as accurately as possible.

We continue by defining the notion of clustering, as used throughout this work.

#### Definition 3

(Clustering) A clustering function *c* is a function that takes as input a set of feature vectors and assigns each of them into a particular cluster, i.e., *c* is a mapping from ${\mathbb {R}}^{|N| \times d}$ to $\rightarrow {\mathbb {N}}^{|N|}$. Each element of the resulting vector of integers represents the label of the cluster, assigned to the corresponding row of the input matrix. The number of clusters *k* can be defined upfront, in which case numbers from 1 to *k* serve as labels for the clusters to which the input vectors belong.

We conclude our list of definitions with the notion of community detection.

#### Definition 4

(Community detection) Let *G* represent a network as defined above. A partition of *G* into *n* non-overlapping subnetworks is any set $\{G_1,G_2,\dots G_n\}$, where $G_i$ are subnetworks of *G*. Let *q* represent a mapping from the set of all possible partitions of *G* to the set ${\mathbb {R}}$. We refer to such a function as a *quality function*. Community detection refers to the process of finding *P*(*G*), which is the particular partition of *G* which optimizes (usually maximizes) the value of the quality function *q*.

Note that the definition of *q* (community scoring function) was not explicitly stated, as existing community detection algorithms optimize for different *q*. Examples of well known *q* include modularity (Louvain algorithm) and average description code length (InfoMap).

### Network representation learning

In this section, we discuss the two node embedding methods we used throughout the empirical evaluation. Both methods were re-implemented using efficient libraries for sparse matrix manipulation, which we also consider as added value of this work.

#### Embedding by factorization

Network embedding algorithms map input networks to dedicated vector spaces, where a node’s neighborhood’s properties are kept approximately intact. Since the considered networks do not contain any node or edge features apart from their weights, we consider shallow network embeddings. We first describe NetMF (Qiu et al. 2018), a recently introduced network embedding methodology, which implements the embedding process as implicit matrix factorization. For example, the well known DeepWalk algorithm (Perozzi et al. 2014) was shown to approximate the following matrix:

$$\begin{aligned} M_{DeepWalk} = \log \bigg (\text {vol}(G) \bigg (\frac{1}{T}\sum _{r=1}^{T} (D^{-1} \cdot A )^{r} \bigg )\cdot D^{-1} \bigg ) -\log b; \end{aligned}$$

where *T* is the context window size, *b* the number of negative samples, $D = diag(d_1,\dots ,d_{|N|})$, where $d_i$ represents generalized degree of node *i* and *A* the adjacency matrix, and *vol*(*G*) is the volume of a weighted graph, defined as: $\sum _{i,j} A_{i,j}$. Such network embedding ideas originate from the initial word representation learner word2vec (Mikolov et al. 2013). We re-implemented NetMF in PyTorch, as the original version was implemented in the now deprecated Theano library (Bergstra et al. 2010). We refer the interested reader to the original paper for theoretical details regarding the method (Qiu et al. 2018).

#### Embedding by personalized node ranking

The other node embeddings we test are Personalized PageRank vectors, obtained by the Personalized PageRank with Shrinking algorithm, recently introduced as part of HINMINE methodology (Kralj et al. 2018). Here, vectors representing personalized node ranks are computed using the power iteration discussed next, whose output consists of P-PR vectors.

For each node $u \in V$, a feature vector $\gamma _u$ (with components $\gamma _u(i)$, $1\le i \le |N|$) is computed by calculating the stationary distribution of a random walk, starting at node *u*. The stationary distribution is approximated by using power iteration, where the *i*-th component $\gamma _{u}(i)^{(k)}$ of approximation $\gamma _u^{(k)}$ is computed in the $k+1$-st iteration as described below. The number of iterations *k* is increased until the stationary distribution converges to the stationary distribution vector (P-PR value for node *i*).

2 $$\begin{aligned} \gamma _{u}(i)^{(k+1)} = \alpha \cdot \sum _{j \rightarrow i}\frac{\gamma _{u}(j)^{(k)}}{d_{j}^{out}}+(1-\alpha ) \cdot v_{u}(i);k = 1, 2,\dots \end{aligned}$$

In Eq. 2, $\alpha$ is the damping factor that corresponds to the probability that a random walk follows a randomly chosen outgoing edge from the current node rather than restarting its walk. The summation index *j* runs over all nodes of the network that have an outgoing connection toward *i* (denoted as $j \rightarrow i$ in the sum), and $d_{j}^{out}$ is the out degree of node $d_{j}$. The term $v_{u}(i)$ is the restart distribution that corresponds to a vector of probabilities for a walker’s return to the starting node *u*, i.e. $v_{u}(u) = 1$ and $v_u(i)=0$ for $i\ne u$. This vector guarantees that the walker will jump back to the starting node *u* in case of restart^1^ (Page et al. 1999). The HINMINE version of this algorithm was additionally parallelized via Multiprocessing library^2^ where 2-4x speedups were observed.

### Clustering of node embeddings

We begin this section with a formal definition of the clustering problem being solved, followed by the considered evaluation of cluster quality. Let emb=*f*(*G*) represent a computed *d*-dimensional node embeddings, thus emb $\in {\mathbb {R}}^{|N| \times d}$. Obtaining a node partition using a clustering algorithm (representing a mapping *c*) of choice can thus be stated as

$$\begin{aligned} P(G) = c(f(G)). \end{aligned}$$

In this work, we consider clustering using efficient miniBatch k-means algorithm (Sculley 2010), which we briefly discuss next. Given a set of row vectors ${\mathcal {X}} \subseteq {\mathbb {R}}^d$, the objective of miniBatch k-means is to find a set *C* of *k* cluster centers $C=\{c_i, \dots ,c_k\}\subseteq {\mathbb {R}}^{d}$ which minimizes the following sum:

$$\begin{aligned} \sum _{r \in {\mathcal {X}}} \min _{c \in {\mathcal {C}}} \big ( \text {dist}(r,c) \big ). \end{aligned}$$

The dist in this work denotes the Euclidean distance ($\Vert r -c \Vert ^{2}$), even though other distances can be used. This problem is NP-hard (Drineas et al. 2004), however it can be approximated well using random cluster *initializations*. In this work, we exploit the k-means++ algorithm for the initialization step [see Arthur and Vassilvitskii (2007) for more details]. The considered miniBatch k-means algorithm also leverages the notion of sparse cluster centres, inspired by the power law nature of word occurrences. Here, the idea is to emphasize the points which occur commonly, as the majority of the points (e.g., words) could be very sparsely distributed and thus contribute little to cluster assignment. One of the reasons we selected this variation of k-means as the clustering detection method is also the similar, heavy tailed nature of node connectivity (Barabási 2009), resembling that of word occurrences. This observation indicates that similar heuristics could perform well. The miniBatch k-means algorithm is thus used to extract *k* clusters from the node embedding space. We next discuss, how to determine whether the *k* clusters represent a good partition.

#### Estimating cluster quality with Silhouette score

The Silhouette score was initially introduced in Rousseeuw (1987). The score has been successfully used to develop novel categorical data clustering algorithms (Aranganayagi and Thangavel 2007), as well as text for clustering tasks (Hotho et al. 2002), and can be defined as follows. Assume the input data was clustered into *k* distinct clusters. The average distance between a given point *i*, and the remainder of the cluster is computed as:

$$\begin{aligned} a(i) = \frac{1}{|C_{i}|-1} \sum _{j\in C_i}\text {dist}(i,j); \end{aligned}$$

where $C_i$ is the cluster to which *i* belongs, and distance $\text {dist}$ is defined by the user. In this work, we employ the Euclidean distance, thus computing $d(i,j) = \Vert i - j \Vert ;$.

The second part of the Silhouette score estimates the dissimilarity with other clusters as follows:

$$\begin{aligned} b(i) = \min _{i \ne j} \frac{1}{|C_j|}\sum _{k \in C_j}\text {dist}(i,k); \end{aligned}$$

thus computing the smallest average distance of *i* to the points of the cluster $C_j$. The Silhouette of a single point can be defined as:

$$\begin{aligned} s(i) = \frac{b(i) - a(i)}{\max {[a(i),b(i)]}}; \end{aligned}$$

where we additionally define $s(i)=0$ if $|C_i|=1$ (when *a*(*i*) is not defined). Note that the *s*(*i*) falls in the interval $[-1,1]$. Intuitively, Silhouette values near $-1$ represent non-distinct clustering, values around 0 represent overlapping clusters and higher values represent more defined clusters. Finally, estimating the global clustering translates to averaging the Silhouette score across the points of interest, as follows:

$$\begin{aligned} \text {Silhouette}(P(G)) = \frac{1}{|N|}\sum _{i \in N} s(i). \end{aligned}$$

Obtaining the Silhouette score for each node thus corresponds to the estimate of how well a given node is clustered, whereas averaging the scores across the considered partition *P*(*G*) gives an estimate of the *global* clustering quality score.

#### SCD formulation

We discussed first how the embedding space of nodes can be subject to *k*-means clustering, yielding potential node partition. Second, we showed how a given partition can be evaluated in terms of intra- and inter- cluster homogeneity via the Silhouette score. The missing part to be discussed in this section is the formal statement of the optimization problem at hand, as well as the numeric procedure used to derive the final *k*.

For readability purposes, we define as $\text {Silhouette}_{\text {G}}(k)$ the Silhouette score obtained using a given *k* (parameter of the k-means algorithm). We thus assume the network node embeddings were obtained from *G* before running the clustering algorithms. The proposed embedding-based Silhouette Community Detection (SCD) algorithm, summarized in Algorithm 1 works as follows.

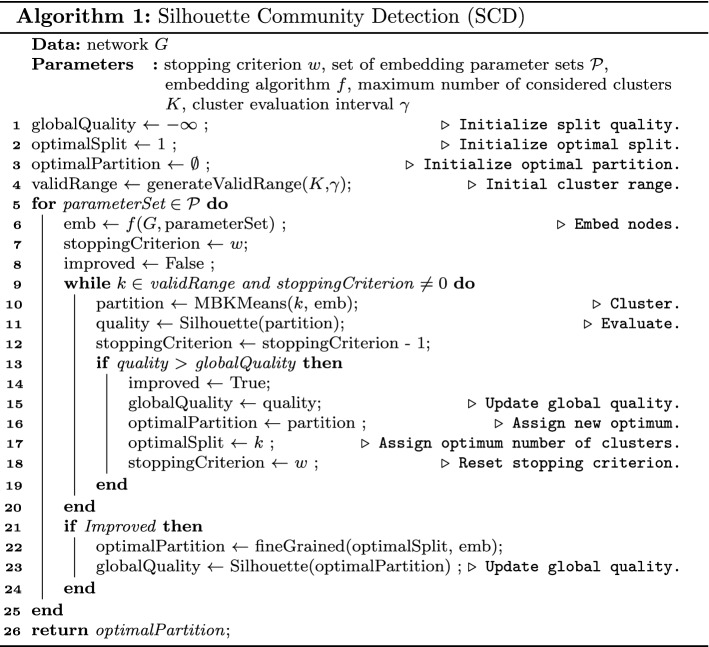



The algorithm traverses the space of embeddings of interest (${\mathcal {P}}$). For each embedding, computed using an embedding procedure *f*, a parameter sweep across values of *k* is conducted. SCD employs a two-step approach to finding the optimal *k*. First, it traverses *k* values defined as part of the validRange—an interval of potential Silhouette optima. This range of *k* values is initially determined based on *K*, the maximum number of clusters to be considered, and $\gamma$, an interval of *k* values being considered. We further demonstrate how $\gamma$ can be *automatically* determined based on *K* in Sect. 3.4. We define this interval as equally distributed natural numbers, where the distance between the numbers is uniformly distributed (e.g., we take every 10-th number on the interval between 1 and 1,000). If the global Silhouette is improved during this parameter sweep (MBKMeans represents the miniBatch k-means algorithm and Silhouette the computation of a given partition’s score), the *k*, as well as the exact partition are stored.

The second step of finding the optimal *k* is a fine-grained optimization step (fineGrained, line 22). Here, the neighborhood of the previously identified *k* (elements of validRange) is explored in more detail—an interval around the *k* is exhaustively inspected. We additionally introduced a stoppingCriterion parameter, which stops the optimization, if Silhouette is not improved in *w* iterations. Once the SCD concludes, it yields the partition of the nodes (or elements of the vector space) into a finite set of communities.

To address the problem of finding the optimal *k*, we thus consider the following steps. First, the space of possible *k* values is not densely defined i.e. we test only every *n*-th *k*. Second, we introduce a stopping criterion—when no improvement is made for enough updates, the algorithm starts a fine-grained search around a currently optimal *k* identified as part of the initial, coarse-grained *k* sweep.

### Formal analysis

In this section we overview and summarize the key parts of the proposed Silhouette community detection algorithm. We begin by formulating the optimization problem that is being solved, followed by the analysis of the relevant aspects of the computational complexity.

Let ${\mathcal {P}}$ represent the set of the node embedding parameters, ${\mathcal {K}}$ the set of candidate *k* values representing the number of clusters. $\text {SilhouetteGlobal} (p,k)$ represents the graph *G*’s partition scored with a Silhouette score obtained when parameter set *p* was used along with *k* clusters to obtain communities. The proposed Silhouette community detection algorithm thus attempts to solve the following optimization problem:

$$\begin{aligned} P_{\text {final}}(G) = \mathop {\hbox {arg max}}\limits _{k \in {\mathcal {K}},p \in {\mathcal {P}}} \big [ \text {SilhouetteGlobal} (k,p) \big ]. \end{aligned}$$

As the quality of the obtained communities depends on the clustering as well as the embedding algorithm, we then discuss the computational complexity of the two steps.

First, contemporary node embedding algorithms can perform in subquadratic time with respect to the number of nodes and have spatial complexity, which is linear with respect to the number of edges. The *k*-means clustering family of algorithms is quadratic in the worst case, yet, the miniBatch sparse version used in this work in practice performs very fast, as it takes the sparsity of the input space into account. Its complexity is ${\mathcal {O}}(\phi \cdot k \cdot |N| \cdot d)$ for a given *k*, where $\phi$ corresponds to the number of steps required by k-means++ initialization.

Second, the Silhouette computation can be performed in ${\mathcal {O}}(|N|^{2} \cdot d)$ time, indicating that the dimension of the embedding plays an important role in the performance of this final step. As one of the main bottlenecks of the proposed method, we recognize the number of cluster evaluations. Thus, the validRange method, discussed in Algorithm 1, can contribute notably to the execution time (values of *k* considered). The total computational complexity of the approach is thus ${\mathcal {O}}(\phi \cdot k \cdot |N|^{2}\cdot d)$. As shown in the following sections, node embeddings are in practice computed less frequently than the clustering, rendering the method more sensitive to the *k* parameter than to the embedding setting considered. Finally, as the dimensionality *d* of the embedding can vary from as little as 5 [hyperbolic embeddings (Nickel and Kiela 2017)] to as much as 1000 or more, we note that selecting the sufficient (and low-dimensional) network embedding can offer realizable speedups of several orders of magnitude.

Finally, we analytically derive an estimate for $\gamma$, the size of the *k* sampling interval with respect to the maximum number of communities expected (*K*) as follows:

$$\begin{aligned} \gamma = \root 3 \of {K^{2}} \end{aligned}$$

For readability purposes, we omit the derivation of this estimate to “Appendix 1”. Note that the rationale behind introduction of this estimate is performance, as compared to the worst case, where *K* different cluster sizes are considered, here we consider a substantially lower number and thus speed up the cluster detection process (this can result in an order of magnitude speedups). In the following sections, we discuss the empirical setting, used to evaluate the performance of the proposed embedding-based SCD algorithm.

## Quantitative evaluation of SCD

This section addresses quantitative evaluation of the proposed embedding-based SCD algorithm. In Sect. 4.1 we present the experimental setting, followed by the results of comparison with state-of-the-art methods in Sect. 4.2.

### Experimental setting

This section presents the empirical evaluation setting used to assess the performance of the proposed SCD approach. We first discuss the baseline network community detection methods, and continue with the description of the networks the methods were tested on.

#### Considered algorithms

We tested three community detection algorithms, and two variants of SCD.

**Baselines.** We compare the proposed SCD approach against the following methods:

- **InfoMap** (Rosvall et al. 2009b). This information flow-based algorithm represents a gold standard for community detection task.
- **Louvain algorithm** (Clauset et al. 2004). Similarly to InfoMap, Louvain algorithm is one of the most widely used community detection algorithms.
- **Label propagation** (Cordasco and Gargano 2010). This simple baseline propagates the information in a breadth-first type of manner and serves as a weak baseline.

**SCD implementations tested.** We tested two implementations of SCD, based on representations, obtained by two network embedding algorithms; namely:

- **SCD-NetMF**. The Silhouette score optimization is conducted based on node representations obtained by the NetMF approach, which we re-wrote in PyTorch (Paszke et al. 2017) for the purpose of this work.
- **SCD-PPR**. The Personalized PageRank with Shrinking algorithm (PPR) is used to obtain stationary distributions of random walkers, representing a series of features for each node. The implementation is based on the one used by the HINMINE algorithm, introduced in Kralj et al. (2018). We used the version of the HINMINE algorithm which was further parallelized in Škrlj et al. (2018).

#### Synthetic networks considered

In this section, we discuss the networks we used for empirical evaluation of the proposed approach. We conducted benchmarks over a space of synthetic Lancicinetti-Fortunato-Raddichi (LFR) networks (Lancichinetti et al. 2008). This family of network models generates networks with corresponding ground truth communities. Such networks are commonly used to evaluate the community detection properties over a larger space of graphs with diverse topological properties (Lancichinetti and Fortunato 2009; Yang et al. 2016).

The considered LFR networks are determined by the following parameters:

- Total number of nodes.
- Average node degree.
- Maximum node degree.
- Mixing. This parameter determines how well defined the generated communities are. It spans from 0 (very well defined) onwards, where, for example, graphs with mixing=1 have very poorly defined communities.
- Degree exponent. Exponent of the node degree distribution (e.g., 2 implies power law network)
- Community exponent. Exponent determining the community sizes.

We generated the space of networks defined by combinations of the following parameters:

- Numbers of nodes:: [100,500,750,1000,2500,5000,10000]
- Average node degrees: [15,30,50]
- Maximum node degrees: [10,50,100,500]
- Mixing: [0.1,0.2,0.5,0.7,0.9]
- Degree exponent: 2
- Community exponent: 1

**Fig. 3:**
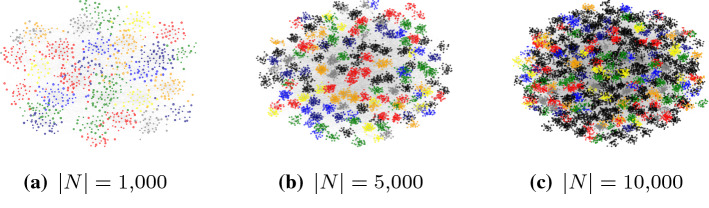
Three examples of LFR networks. The largest example LFR network consists of 10,000 nodes and 302,160 edges. The mixing parameter for these networks is set to 0.1, indicating very well defined communities. We colored the first 100 communities by size (random colors)

In total, we generated 234 valid networks with various topological properties. Example LFR network with highlighted communities is shown in Fig. 3

#### Real social network used

**Fig. 4:**
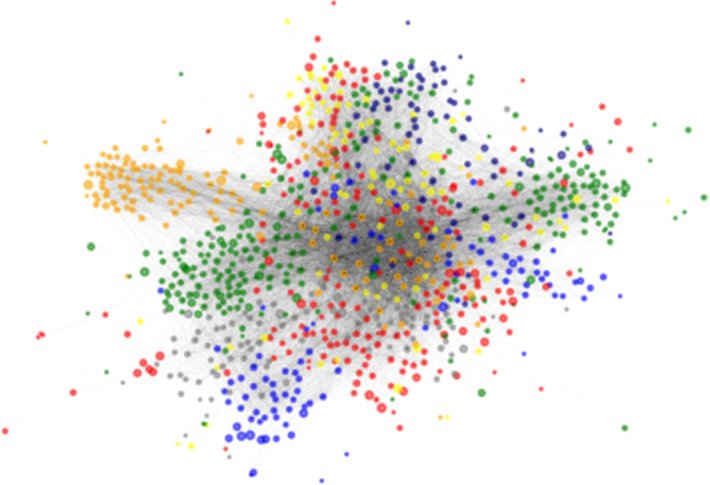
E-mail ground truth communities (departments of senders)

Further, we test how well communities can be detected on a network with known ground-truth communities corresponding to E-mail network of one of the large European research institutions (Yin et al. 2017). An edge (u, v) in the network denotes that the person u sent person v at least one email. The e-mails only represent communication between institution members (the core), and the data set does not contain incoming messages from or outgoing messages to the rest of the world. The network consists of 1005 nodes and 25,571 edges, and is, along with its ground truth communities, visualized in Fig. 4. The data set also contains “ground-truth” community memberships of the nodes. Each individual belongs to exactly one of the 42 departments at the research institute.

#### Technical details

In this section, we discuss some hardware-specific implementation details. The machine the benchmarks were run on was a Intel(R) Xeon(R) Gold 6150 CPU @ 2.70GHz processor equiped machine with 64GB of RAM. The machine also has a Nvidia Tesla GPU, which we used to test whether our NetMF implementation works as expected (on GPU). For the actual benchmarks, *we did not use* GPU for factorizing the network, in order to more easily compare the execution times on CPU only. We intentionally didn’t use GPU to demonstrate that no specialized hardware is needed to obtain competitive results. The LabelPropagation baseline was implemented using Hagberg et al. (2008), the Louvain algorithm implementation, as well as a wrapper for the InfoMap binary can be found in Skrlj et al. (2019c), Škrlj et al. (2019b). The validRange for the empirical evaluation was set to the interval [5, |*N*|, 10]. The embedding space parameters used during optimization were: number of negative samples ($\{1,5,20\}$), window size ($\{1,3,5,10,30,50\}$) and embedding dimension ($\{16,32,64,128,256\}$). In “Appendix 2”, we explain how the social network’s Silhouettes were normalized based on the level of embedding dimension which yielded more robust results.

#### Community quality evaluation measures

An established approach to evaluating the quality of community detection algorithms is via *ground truth communities*. We assume the “optimal” partition *P*(*G*) (ground truth) is known upfront. A partition similarity score (as defined next) is used to compare the ground truth partition with the one returned by a community detection algorithm.

We next discuss the measures of performance we used to evaluate how well a given algorithm is able to detect communities. We employ the following three measures. **Normalized Mutual Information (NMI)** This index is defined as follows:

$$\begin{aligned} \text {NMI}(Y,C) = \frac{2 \times I(Y;C)}{H(Y) + H(C)}; \end{aligned}$$

where *H*(*C*) denotes the entropy of the assigned labels *C*, the *H*(*Y*) entropy of the ground-truth labels *Y*. The *I*(*Y*; *C*) denotes the mutual information (Thomas and Cover 1991; Toni et al. 2009) between *Y* and *C*. Thus, the larger the score, the better the matching.

**Adjusted Rand Index (ARI)** This index measures a similarity between two clusterings by considering all pairs of samples and counting pairs that are assigned in the same or different clusters in the predicted and true clusterings. For readability purposes we do not define it here, yet we refer the reader to Rand (1971) for the exact formulation.

**Modularity** The modularity measure (Clauset et al. 2004) is defined for a network partitioned into communities as follows:

$$\begin{aligned} Q = \frac{1}{2m}\sum _{v=1}^n\sum _{w=1}^n \bigg [ A_{v,w} - \frac{k_{v}k_{w}}{2m} \bigg ]\delta (c_{v},c_{w}); \end{aligned}$$

where *n* represents the number of nodes and *m* the number of edges, $[A_{v,w}]_{v,w=1}^n$ denotes the adjacency matrix (i.e. $A_{v,w}$ is 1, when *u* and *v* are connected by an edge, and 0 otherwise), $k_{v}$ denotes the degree of the *v*-th node and $c_v$ denotes the community the *v*-th node is assigned to. The $\delta (c_{v},c_{w})$ represents the Krönecker delta function, which amounts to 1 when $c_v=c_w$ and 0 otherwise. The value $\frac{k_{v}k_{w}}{2m}$ represents the average fraction of edges between nodes *v* and *w* in a random graph with the same node degree distribution as the considered graph. Note that some of the baseline methods (e.g., Louvain algorithm) directly optimize the modularity, and are thus expected to perform favorably with respect to this metric. However, it was shown for modularity to have a resolution limit—it may not be able to detect smaller communities, even if they are apparent (Good et al. 2010; Zhang et al. 2009; Fortunato and Barthelemy 2007). One of the purposes of the conducted experiments is to further explore and confirm the claim that even if the modularity is high, the communities are not necessarily well detected.

### Experimental results

In this section, we present the results of the empirical evaluation, starting with the results obtained on synthetic benchmark networks in Sect. 4.2.1, and followed by the results on the real-world social network in Sect. 4.2.2.

#### Results on synthetic networks

Analyzing the 2-step search for the optimal value of *k* in Algorithm 1, we observe the proposed optimization in majority of cases finds a sufficient optimum—once the local optimum is found, some additional steps are performed to evaluate whether there exists a better solution in close range. In this work, we do not focus extensively on estimating the initial range of *k*, yet we believe such estimations could offer potentially better detection.

**Fig. 5:**
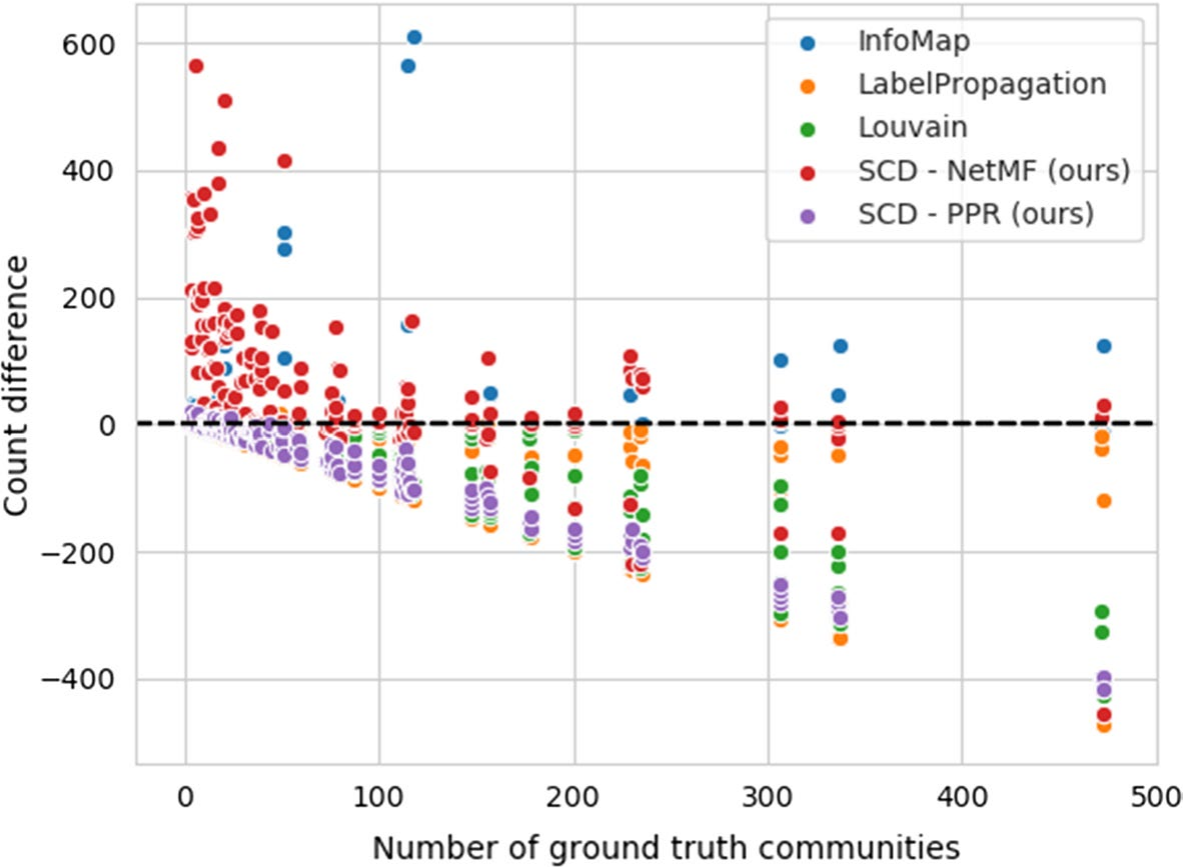
Differences in the number of estimated communities (on LFR networks). The horizontal line represents optimal outcome with respect to the number of detected communities

The visualization of overall differences between the number of estimated communities and the number of ground-truth ones is shown in Fig. 5—the horizontal line represents the perfect match in the number of detected with that of ground truth communities. We can observe that the proposed SCD overestimates the number of communities when small number of ground truth communities is present. However, the numbers stabilize when more than 100 communities are present. On the other hand, we can observe larger deviations with Louvain algorithm when the larger number of communities are present, indicating that Louvain algorithm algorithm under-estimates the number of communities. Similarly to SCD, InfoMap also over-estimates the number of communities when many communities are present, but for large numbers of ground truth communities, the over-estimation is more evident in InfoMap.

We believe performance with respect to the mixing parameter determining LFR graphs is of crucial importance, as it offers insight into how the considered community detection algorithms behave when communities are more or less defined. We show overall results, summarized with respect to this aspect in Table 1.

**Table 1:** Results over the space of synthetic networks

Mixing	Algorithm	NMI	ARI	Modularity
0.1	InfoMap	**0.994** ± **0.028**	**0.996** ± **0.02**	0.816 ± 0.118
0.1	LabelPropagation	0.988 ± 0.038	0.96 ± 0.075	0.81 ± 0.133
0.1	Louvain algorithm	0.98 ± 0.031	0.925 ± 0.115	**0.817** ± **0.115**
0.1	SCD-NetMF (ours)	0.96 ± 0.089	0.924 ± 0.122	0.754 ± 0.195
0.1	SCD-PPR (ours)	0.936 ± 0.051	0.867 ± 0.114	0.364 ± 0.307
0.2	InfoMap	0.922 ± 0.359	**0.939 ± 0.223**	0.698 ± 0.181
0.2	LabelPropagation	0.743 ± 0.961	0.832 ± 0.259	0.674 ± 0.217
0.2	Louvain algorithm	**0.969** ± **0.057**	0.898 ± 0.136	**0.713** ± **0.127**
0.2	SCD-NetMF (ours)	0.949 ± 0.11	0.911 ± 0.151	0.662 ± 0.174
0.2	SCD-PPR (ours)	0.907 ± 0.09	0.815 ± 0.154	0.305 ± 0.269
0.5	InfoMap	0.715 ± 0.903	**0.848** ± **0.32**	0.411 ± 0.152
0.5	LabelPropagation	0.378 ± 1.174	0.398 ± 0.3	0.326 ± 0.21
0.5	Louvain algorithm	0.846 ± 0.238	0.657 ± 0.274	**0.435 ± 0.102**
0.5	SCD-NetMF (ours)	**0.856** ± **0.197**	0.75 ± 0.278	0.377 ± 0.137
0.5	SCD-PPR (ours)	0.75 ± 0.233	0.502 ± 0.273	0.157 ± 0.179
0.7	InfoMap	0.498 ± 1.135	**0.471** ± **0.467**	0.166 ± 0.134
0.7	LabelPropagation (*)	**0.679 ± 3.188**	0.0 ± 0.0	0.0 ± 0.0
0.7	Louvain algorithm	0.648 ± 0.293	0.325 ± 0.241	**0.262** ± **0.04**
0.7	SCD-NetMF (ours)	0.492 ± 0.306	0.258 ± 0.367	0.118 ± 0.103
0.7	SCD-PPR (ours)	0.407 ± 0.233	0.119 ± 0.195	0.03 ± 0.055
0.9	InfoMap	**0.493** ± **2.529**	0.001 ± 0.012	0.031 ± 0.051
0.9	LabelPropagation (*)	0.744 ± 3.196	0.0 ± 0.0	0.0 ± 0.0
0.9	Louvain algorithm	0.052 ± 0.024	0.002 ± 0.003	**0.164 ± 0.043**
0.9	SCD-NetMF (ours)	0.209 ± 0.109	0.003 ± 0.007	0.024 ± 0.03
0.9	SCD-PPR (ours)	0.193 ± 0.176	**0.019** ± **0.077**	0.008 ± 0.024

Values represent averages for each mixing level across other parameters. We highlight top performers (first and second), with respect to NMI, as well as ARI scores. The LabelPropagation algorithm detects communities at higher mixing very poorly, we marked such runs with “*”

The best results for each mixing value are bolded

The results offer insights into performance of different algorithms with respect to various measures. As expected (and discussed in Sect. 6), the modularity score, which is optimized by the Loivain algorithm, is the highest with this algorithm. Other algorithms interchangeably outperform one another, indicating optimizing different metrics potentially leads to specialization in different parts of the networks space, thus leading to performance trade-offs.

#### Results on a real-world network

We also test the performance on a real-world E-mail network with known ground truth communities.

**Table 2:** Community detection results on a real-world network representing E-mail senders. The LabelPropagation algorithm was not able to detect any communities, values of all scores were lower than $10^{-6}$

Algorithm	NMI	ARI	Modularity
InfoMap	0.654	0.301	0.39
Louvain algorithm	0.577	0.300	**0.41**
LabelPropagation	$< 10^{-6}$	$< 10^{-6}$	$< 10^{-6}$
SCD-PPR	0.552	0.235	0.31
SCD-NetMF ($d = 128$)	**0.720**	0.437	0.330
SCD-NetMF ($d = 32$)	0.711	**0.462**	0.342

The best results for each mixing value are bolded

**Fig. 6:**
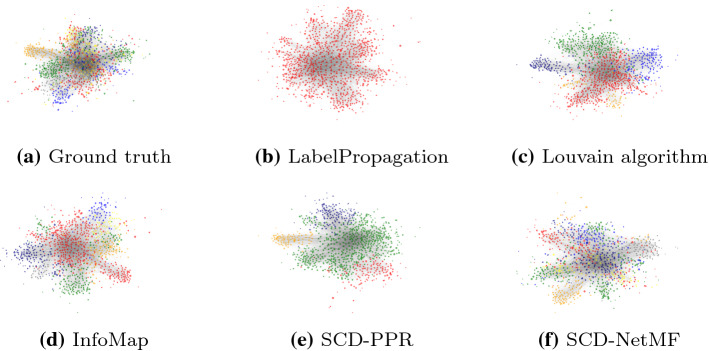
Visualization of E-mail network communities obtained using different algorithms. Communities are colored by size. The LabelPropagation and SCD-PPR performed the worst, which is also apparent from the visualizations—LabelPropagation did not detect any communities, whereas SCD-PPR detected too few

The proposed SCD approach shows best performance on the mentioned real-life network (Table 2). The best performing embedding w.r.t NMI score was of dimension 128, with negative sampling parameter set to 1 and window size of 5. Interestingly, we also state the performance of a much smaller embedding ($d=32$), where the ARI score was better than in the case of the larger-dimensional one. Here, negative sampling was set to 1 and window size to 3. This result indicates that even embeddings of lower dimensionality potentially capture enough node similarity information that they are successfully grouped into communities. We additionally visualize the results using the Py3plex (Škrlj et al. 2019b) library in Fig. 6. The colors are based on community sizes—when communities are obtained (or given), they are sorted by size and colored with a predefined set of colors. Thus, the top three communities by size are colored red, green and blue. InfoMap, as well as SCD-NetMF ($d=128$) approaches detected the largest ground truth community, which is originally present in two parts (Fig. 6a), and as such harder to detect.

**Fig. 7:**
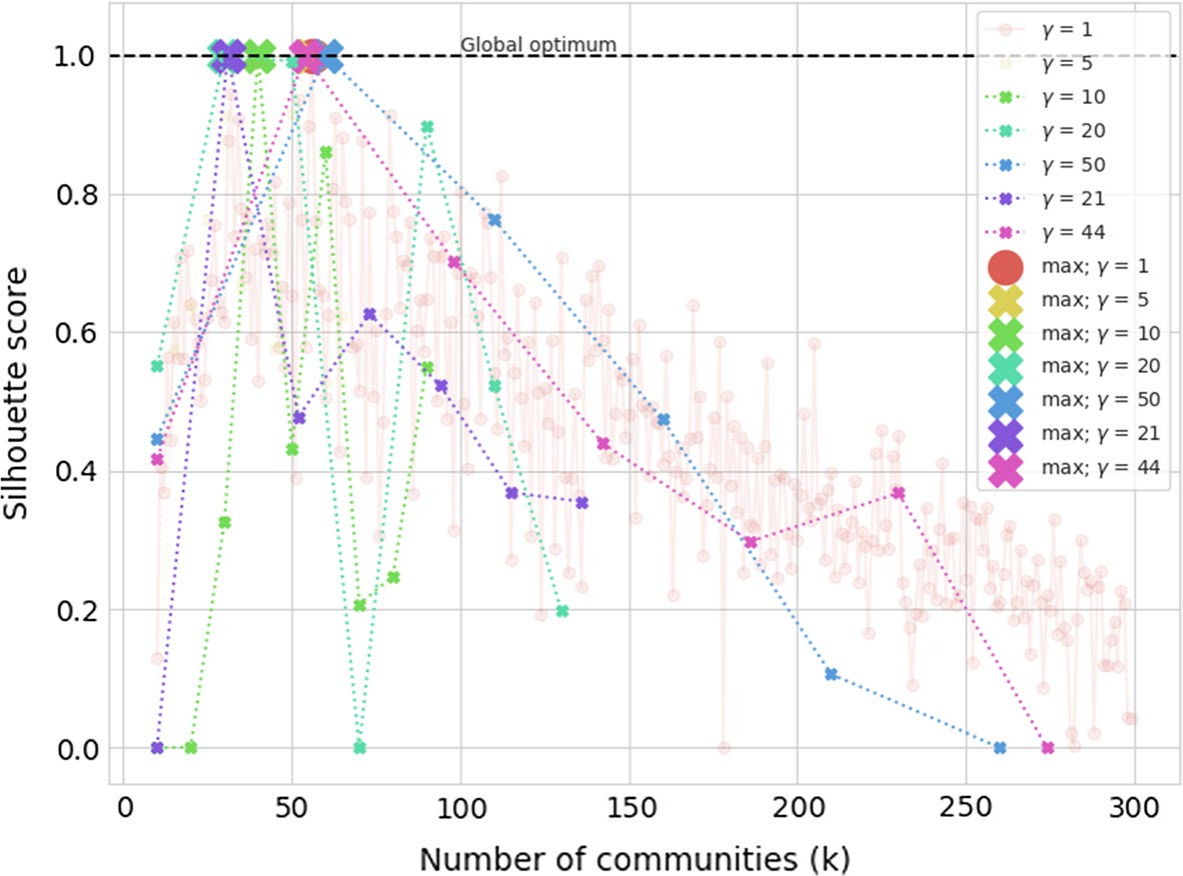
Visualization of solutions found when different intervals of *k* are considered. The situation where each *k* is tested corresponds to $\gamma = 1$. The larger markers denote optima found when different intervals of *k* are considered. It can be observed, all $\gamma$ variants yield similar number of communities ($\approx 50$), which is close to the ground truth of 42 communities

Similarly to the results obtained on synthetic networks, the Louvain algorithm, InfoMap and SCD-NetMF perform well—the communities they emit are visually distinct and resemble the ground truth network’s ones. We can also observe SCD-PPR identified fewer communities compared to other approaches, indicating that taking whole stationary distributions into account is potentially not optimal.

We next present the results of exhaustive empirical validation of the proposed Silhouette optimization procedure. For this task, we used the real-world social network discussed previously, and explored how the results of the proposed optimization correspond to a situation, where each *k* is computed (exhaustive evaluation $\gamma =1$). The results for different values of *k* are visualized in Fig. 7. Here, we normalized individual Silhouette scores obtained for different $\gamma$ values for readability purposes.

It can be observed that when different intervals of *k* considered (denoted as *k* in the Fig. 7) yield very similar results, where the number of communities is between 30 and 50 (number of ground truth communities is 42). This result indicates that the proposed SCD is not sensitive to $\gamma$, indicating potential speedups can be obtained, should this interval be selected based on e.g., a given network’s properties—such situations are shown with pink ($K=300$) and violet ($K=100$), indicating the proposed $\gamma$ estimation offers good results. Note that individual SCD runs were for this figure run with stopping parameter set to 5—if after 5 iterations no improvement was made, the current optimum was returned as the result. The global Silhouette optimum (red points, indicating exhaustive search) was when $k=54$.

## Qualitative evaluation of SCD: Community explanation

In this section we discuss how the obtained communities can be combined with background knowledge in the form of ontologies to provide human-understandable rules, describing individual communities.

### Methodology

This section discusses how the in-house CBSSD methodology can be employed for obtaining such descriptions (Škrlj et al. 2019a) (see Sect. 2 for more details). We first introduce the notions of subgroup and semantic subgroup discovery. Next, we discuss how communities can be understood as target classes for the task of semantic subgroup discovery. We also describe Hedwig, a semantic rule learner, which was for the purpose of this work parallelized to scale to thousands of candidate communities. In the following sections we discuss the key ideas of semantic subgroup discovery, as used in the remainder of this work.

#### Parameters of the Hedwig semantic subgroup discovery algorithm

We next discuss how the task of semantic subgroup discovery was performed on obtained communities. For this task, we consider the Human Affinome, a collection of empirical protein interactions curated for the Affinomics consortium. The parsed graph consists of 1171 nodes and 1571 edges, with the average degree of 2.68. The same grid-based search for the maximum Silhouette as in the benchmark experiments (see Sect. 4.1.4) was used. The following parameters were set for Hedwig—semantic subgroup discovery:

- The alpha value for determining rule significance was set to 0.05
- FDR correction was used, where the threshold was set to 0.1
- Minimum support required was 0.01
- Beam size of 30 was used
- Depth was set to 10

The background knowledge considered was the whole gene ontology (Ashburner et al. 2000), comprised, at the time of writing, of more than 40,000 terms.

As evaluation of rules for each community (separately) would be too time consuming, we selected the communities with the longest term conjuncts, as well as the most significant rules and performed literature-based evaluation of rules. As the discovered term conjuncts possibly represent well known biological interactions, we investigated individual rules separately, and compared their conjuncts to descriptions of genes, present in the studied community.

#### Technical details: speeding up Hedwig

We next discuss the improvements we made to the original Hedwig algorithm for it to handle larger collections of background knowledge and hundreds of target classes (as required in this work)

As the number of communities can be in the order of hundreds (or even thousands), one needs to consider |*P*| [the number of communities) individual learners. As shown in Škrlj et al. (2019a)], this step can be time consuming, which made us realize that implementing Hedwig in parallel can reduce the time overhead. The implementation used in this work was parallelized at the class level. Here, we recognized as independent each class-specific learner. Thus, depending on the number of available CPU cores, Hedwig considers multiple communities simultaneously, offering from 5x to 15x speedups when compared to the original single CPU version.

### Results of qualitative analysis

**Fig. 8:**
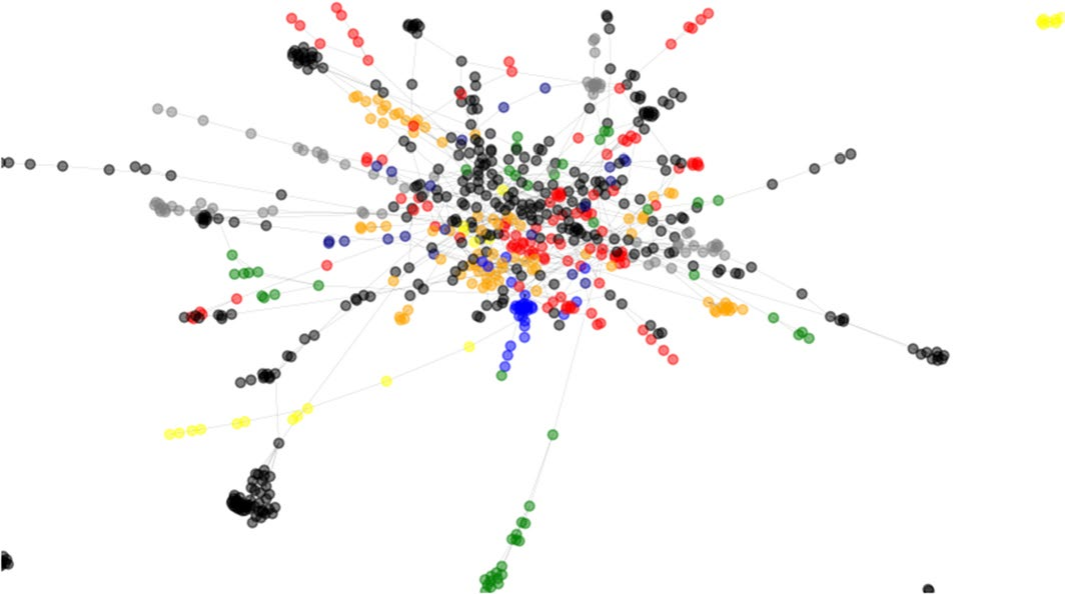
Communities in the Human Affinome network

In Sect. 4.2 we demonstrated the overall community detection performance of the proposed SCD. In this section we show the results of semantic subgroup discovery on the Affinome protein interaction network. This network was obtained based on extensive experimental evidence, and consists of interactions related to core metabolism. The communities, detected in the Affinome are shown in Fig. 8.

**Table 3:** Results of subgroup discovery for the selected community. Section 5.2.2 discusses the meaning of the resulting GO terms with respect to the members of the resulting community

Conjuncts	#Positive	Precision	Lift	Corrected p-value
GO:0007411	8	0.286	31.286	< 0.001
GO:0038128	6	0.316	34.579	< 0.001
GO:0007173	6	0.240	26.280	< 0.001
GO:0050900 $\wedge$ GO:0005515	6	0.188	20.531	< 0.001
GO:0050900	6	0.167	18.250	< 0.001
GO:0038096 $\wedge$ GO:0005515	5	0.208	22.813	< 0.001
GO:0038096	5	0.200	21.900	< 0.001
GO:0001784	4	0.286	31.286	< 0.001
GO:0008286	4	0.267	29.200	< 0.001
GO:0038095	5	0.147	16.103	< 0.001
GO:0005070	3	0.273	29.864	< 0.001
GO:0016303 $\wedge$ GO:0005515	3	0.250	27.375	< 0.001
GO:0008360 $\wedge$ GO:0005886	3	0.214	23.464	< 0.001
GO:0016303	3	0.200	21.900	< 0.001
GO:0007169 $\wedge$ GO:0005524	3	0.188	20.531	< 0.001
GO:0007265	3	0.150	16.425	0.001
GO:0008360	3	0.143	15.643	0.001
GO:0007169	3	0.143	15.643	0.001
GO:0005884	2	0.200	21.900	0.003
GO:0014065	2	0.200	21.900	0.003
GO:0031234 $\wedge$ GO:0004713	2	0.182	19.909	0.004
GO:0071902	2	0.167	18.250	0.005
GO:0097110	2	0.167	18.250	0.005
GO:0004715 $\wedge$ GO:0004713	2	0.167	18.250	0.005
GO:0005901	2	0.143	15.643	0.006
GO:0016337	2	0.143	15.643	0.006
GO:0004715	2	0.143	15.643	0.006
GO:0042542 $\wedge$ GO:0005515	2	0.143	15.643	0.006
GO:0031234	2	0.133	14.600	0.007
GO:0042542	2	0.133	14.600	0.007

#### Results overview

The SCD algorithm discovered 145 communities. As the purpose of this work is not to explain every single one, but to demonstrate how they can be explained, we selected the community with the largest number of rules, as well as the larger number of multi-term conjuncts. The rules are summarized in Table 3. The members of the community the rules describe are summarized in Table 4.

**Table 4:** Members of the described community

UniProt ID	Gene	Name
P27986	PIK3R1	Phosphatidylinositol 3-kinase regulatory subunit alpha
P12931	SRC	Proto-oncogene tyrosine-protein kinase Src
P46108	CRK	Adapter molecule crk
Q07889	SOS	Son of sevenless homolog 1
P08631	HCK	Tyrosine-protein kinase HCK
P42338	PIK3CB	Phosphatidylinositol 4,5-bisphosphate 3-kinase
P29353	SHC1	SHC-transforming protein 1
Q13480	GAB1	GRB2-associated-binding protein 1
P01112	HRAS	GTPase HRas
P07333	CSF1R	Macrophage colony-stimulating factor 1 receptor

#### Interpretation of results

This section interprets how the found rules are associated with the members of the enriched community. We systematically describe first what a given rule represents, followed by to what part of the community it maps.

We first observe, that many of the multi-conjunct rules contain the term “GO:0005515”, which corresponds to “protein binding”. The emergence of this very general term is expected, as the object of study is a protein interaction network. However, we notice that this term always appears in conjuncts with other terms, such as for example the “GO:0042542” (“response to hydrogen peroxide”), “GO:0016303” (“1-phosphatidylinositol-3-kinase activity”), “GO:0038096” (“Fc-gamma receptor signaling pathway involved in phagocytosis”) and “GO:0050900” (“leukocyte migration”).

We can observe that “GO:0016303”, the term representing 3-kinase activity possibly emerged as the result of both PIK3R1, as well as PIK3CB proteins present in the studied community.

Next, both “GO:0038096”, “GO:0042542”, as well as “GO:0050900” represent events that are commonly present during immune response. We believe the aforementioned terms emerged as a consequence of CSF1R (Macrophage-colony factor receptor), HCK (Tyrosine-protein kinase), as well as SRC (Proto-oncogene tyrosine kinase). The tyrosine kinases transmit signals from cell surface receptors and play an important role in the regulation of innate *immune responses*, including neutrophil, monocyte, macrophage and mast cell functions, phagocytosis, cell survival and proliferation, cell adhesion and migration. In combination with CSF1R the terms indicate the considered community is associated with immune response (Zhu et al. 2014).

We discuss the three most significant terms, namely “GO:0007411” (“axon guidance”), “GO:0038128” (“ERBB2 signaling pathway”) and “GO:0007173” (“epidermal growth factor receptor signaling pathway”). The CRK protein, present in the considered community is known to regulate cell adhesion, spreading and migration. The “GO:0038128” represents a signaling pathway comprised of tyrosine kinases (present in the considered community). Similarly the association with the “GO:0007173” term related to epidermal growth factor signaling also corresponds to HRAS (GTPase HRas) and other kinases, which are known to play crucial roles during epidermal growth (Rosenberger et al. 2009).

To summarize, the proteins’ functionality is indeed entailed in the obtained set of rules. Even though the considered community consists mostly of signaling and growth-related proteins, the related rules summarize key aspects such as cellular signaling and growth regulation, thus offering a human-interpretable description of the community without the time-consuming manual search.

## Conclusions and further work

In this section we summarize the obtained results and propose the directions for further work.

### Results summary

We observe embedding-based community detection, as proposed in this work offers competitive performance on both synthetic, as well as real networks. One of the key observation is, SCD performs well if the embedding dimension is low (as can be observed from the computational complexity analysis). Thus, we recognize the recent achievements in the field of hyperbolic network embedding as potential further work. The proposed approach was tested with an efficient implementation of k-means clustering, yet, any clustering algorithm could be employed at this stage of community detection. Potentially more efficient alternatives could offer even faster performance.

Speed-wise, SCD-NetMF performed comparably to InfoMap, which was shown to scale to larger networks, even though the LabelPropagation and Louvain algorithm scaled even better. As discussed, the reduction of embedding dimension, as well as potentially less costly score, which is maximized could speed up the computation even further. However, we believe one of the benefits of the proposed SCD is that it can operate on pre-computed embeddings without any additional modification. This way, the complexity reduces to exploration of *k* space, for which we theoretically, as well as empirically proved that it can be explored efficiently.

In terms of performance, the proposed method performs similarly to InfoMap and Louvain algorithm, optimizing a different measure of community quality (in a space where some information on the network structure is lost), potentially opening many new research venues. Even though we explored some parameterizations of the networks, we did not perform exhaustive search over the space of all embeddings, which we believe could potentially offer even better performance (at a significant computational cost).

The SCD also detected communities, which we interpreted using semantic subgroup discovery tool. Even though the aim of this analysis was not to discover novel knowledge, we were able to retrieve some existing (empiricaly proven) connections between proteins present in the same community, indicating such methodology could also offer novel knowledge when applied in a different setting.

### Further work

We believe the conducted series of experiments also demonstrates, that modularity optimization is not necessarily the optimal tactic for finding the best partition. Modularity, although good at capturing densely connected parts of the graph, appears to miss-score the less apparent, but just as important connections.

Recent discoveries in the field of community number estimation also serve as complementary methodology to the proposed SCD. Here, the initial estimate of the number of communities could be notably improved (we employ a rather naïve scheme and do not consider any maximum-likelihood estimation).

*Non-euclidean geometries.* In this work, both the Silhouette computation, as well as the k-means computation were based on non-euclidean distance. Recent advancements in hyperbolic embeddings of real world networks offer novel insights into hierarchical organization underlying many such systems. Both k-means, as well as the Silhouette can be extended to hyperbolic spaces, for example the Poincaré disc, offering a natural extension for working with such non-euclidean embeddings.

*Complementarity with graph-convolutional networks* Finally, we believe the proposed method is complementary to the recently emerging graph convolutional neural network embedding methodology. This branch of algorithms exploits features assigned to nodes (or edges) to obtain better node representations. As the resulting ${\mathbb {R}}^{|N| \times d}$ space is of the same type as considered in this work, we believe the proposed methodology could open an elegant extension to the research of community detection with meta information.

*Theoretical improvements* Note that even though we offer a theoretical estimation of the $\gamma$ parameter, we believe the work could be further improved should e.g., maximum likelihood-based estimation of the number of communities in a given network be considered. Should such estimate be obtained, the space of *k* values to be explored could be drastically reduced.

*Optimizing the embedding space separately* In this work we performed rather naïve sweep through node embedding space in order to identify the configuration, which yielded the best Silhouette score. However, we believe that in certain applications, the node embeddings can be optimized w.r.t. a different task, e.g., classification, thus eliminating such expensive parameter search. We leave exploration of such claims for further work.

*Exploration of low-dimensional embeddings* One of the interest results of this work is the fact that rather low dimensional embeddings (e.g. $d=32$) already yield good results in terms of community detection. We believe this aspect could be further explored, as *d* is directly associated with computational complexity, thus reducing *d* could yield multifold speedups, as well as offer insights into minimal dimension, needed to uncover a given network’s latent structure.

*Feature-rich node embeddings as input* The proposed SCD can cluster any (non-contextual) node embeddings. Thus, the recent body of work focusing on feature-rich networks, which yield real-valued vectors representing nodes can be naturally used with SCD for the task of community detection.

*Exploration of subnetwork clustering* In this work we explored whether nodes can be grouped in a similar manner to that of contemporary community detection algorithms. However, instead of nodes, one can obtain embeddings of whole subnetworks. The proposed SCD can be naturally extended to such scenario, where, for example, very large networks could first be reduced to modular units, and the clustered. We believe this is one of the potential oportunities to scale SCD.

## Availability

The SCD algorithm is freely available to academic users at https://github.com/SkBlaz/SCD

## Figures and Tables

**Fig. 9:**
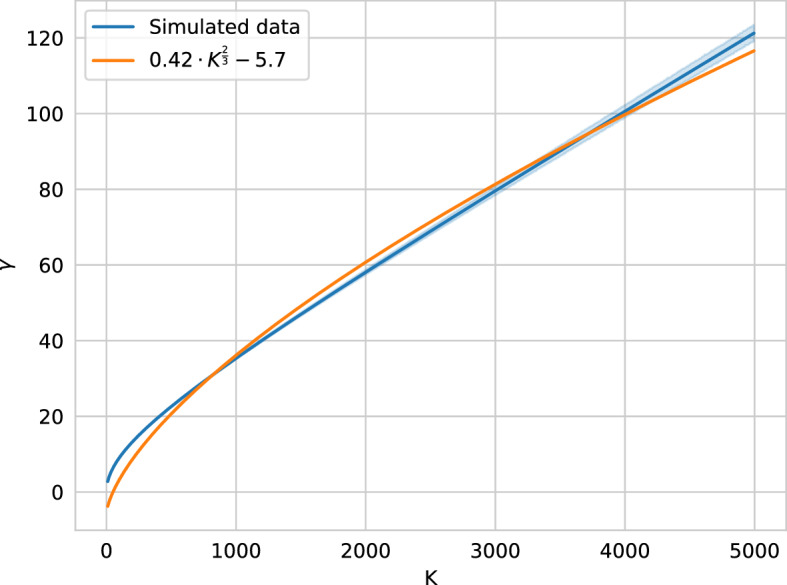
Fitting the closed-form solution for determining the $\gamma$ parameter to simulated data

**Fig. 10:**
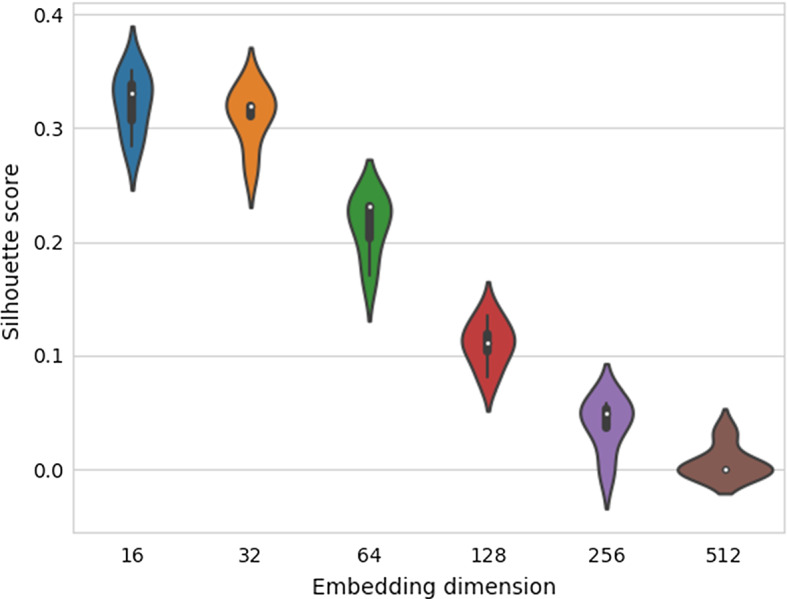
Non-normalized Silhouette accross dimensions on the considered social network

**Fig. 11:**
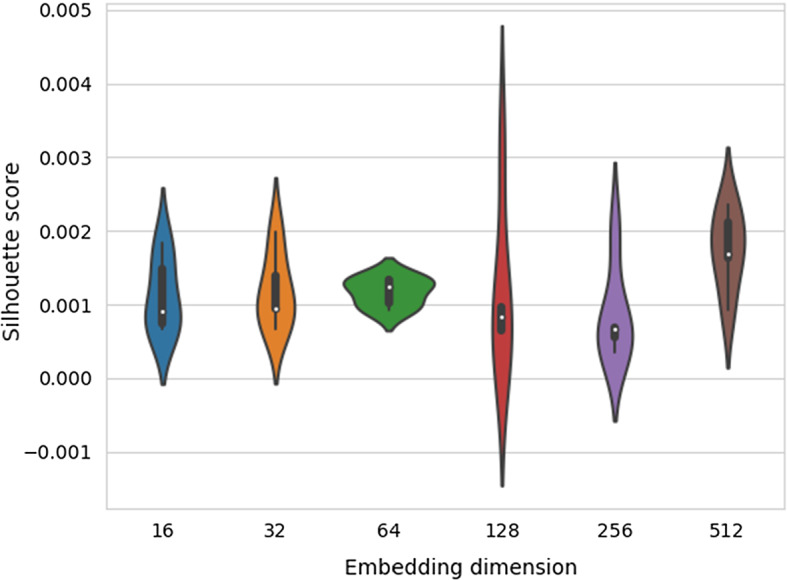
Normalized Silhouette across dimensions on the considered social network

## Appendix 1: Closed-form solution for determining the $\gamma$ parameter

In this section we present the derivation which led to an analytical estimate of the $\gamma$ (the interval in which the number of clusters are considered). We begin by theoretical analysis, followed by evaluation of how well the theoretical estimate fits to a large space of simulated networks.

### Problem statement

The value of the optional parameter $\gamma$ in Algorithm 1 presents a tradeoff between two extremes. On one hand, setting a low value of $\gamma$ means that each value *k* from 1 to *K* must be checked. On the other, a high value of $\gamma$ means that the fineGrained step of SCD (see Algorithm 1, line 22), where a neighborhood of *k*, found in the first part of the Algorithm 1 (lines 9-20) is exhaustively evaluated. For example, let the *k* found in the first part (lines 9-20) be 10 and $\gamma =2$. The fineGrained method of SCD (line 22) additionaly explores the values of Silhouette when $k \in \{9,11\}$—a close neighborhood of the optimum found by the initial *k* search.

To discover the optimal setting of $\gamma$, we first estimate the time complexity of Algorithm 1 when $\gamma =1$. This setting represents the baseline naïve sweep over all candidate values of *k*, and is compared to the search for *k* with a given value $\gamma > 1$.

Without optimizing, with $\gamma =1$, Algorithm 1 does all of the work in its first step. The algorithm calculates *K* Silhouette scores, each with a complexity of ${\mathcal {O}}(|N|^2d)$ and one *k*-means clustering for each $k\in [1..K]$. As the time complexity of the *k*-means clustering is ${\mathcal {O}}(k|N|d)$, the time complexity of this step is ${\mathcal {O}}(|N|d)+{\mathcal {O}}(2|N|d) +\cdots +{\mathcal {O}}(K|N|d) = {\mathcal {O}}(K^2|N|d)$. The entire iteration of Algorithm 1 therefore has a complexity of

$$\begin{aligned} {\mathcal {O}}(K\cdot |N|^2d)+{\mathcal {O}}(K^2|N|d) = {\mathcal {O}}(K|N|d(|N|+K)) \end{aligned}$$

Using a fixed value of $\gamma >1$, on the other hand, means that Algorithm 1 is composed of two phases. In the first phase, values of $k=\gamma , 2\gamma ,\dots , K=\frac{K}{\gamma }\cdot \gamma$ are checked. This means that $\frac{K}{\gamma }$ runs of the Silhouette algorithm and *k*-means clustering for every checked value of *k*, for a total complexity of

$$\begin{aligned}&{\mathcal {O}}\left( \frac{K}{\gamma }|N|^2d\right) + {\mathcal {O}}\left( \gamma |N|d+2\gamma |N|d+\cdots +\frac{K}{\gamma }|N|d\right) \\&\quad ={\mathcal {O}}\left( \frac{K}{\gamma }|N|^2d+\frac{|N|dK^2}{\gamma }\right) = {\mathcal {O}}\left( \frac{K|N|d}{\gamma }(|N|+K)\right) \end{aligned}$$

In the second phase, once the interval holding the optimal value of *k* has been discovered, the $\gamma -1$ possible values of *k* in that interval must still be checked. Assuming that the interval to check is $[n_0\gamma + 1.. (n_0+1)\gamma -1]$, this has a complexity of

$$\begin{aligned} (\gamma -1)|N|^2d + |N|d(n_0\gamma + 1 + n_0\gamma + 2+\cdots + n_0\gamma + \gamma - 1) \\= (\gamma -1)|N|^2d + \left( n_0+\frac{1}{2}\right) (\gamma ^2-\gamma )|N|d \end{aligned}$$

meaning that the total complexity of Algorithm 1’s search for *k* given a fixed value of $\gamma$ is

$$\begin{aligned} |N|d\left( \frac{K(K+|N|)}{\gamma }+|N|(\gamma - 1)+\left( n_0+\frac{1}{2}\right) (\gamma ^2-\gamma )\right) . \end{aligned}$$

This means that the factor by which the time complexity is decreased by using $\gamma$ is equal to

$$\begin{aligned} \frac{1}{\gamma }+\frac{n_0+\frac{1}{2}}{K(K+|N|)}\alpha ^2+\frac{|N|-n_0-\frac{1}{2}}{K(K+|N|)}\alpha -\frac{|N|}{K(K+|N|)}. \end{aligned}$$

The optimal value $\gamma$ at which this factor is minimized can then be obtained as the solution to the equation

3 $$\begin{aligned} - \frac{1}{\gamma ^{2}} + 2 \cdot \frac{1}{\cdot K \cdot (K + |N|)} \cdot \gamma + \frac{k + |N| - 0.5}{K \cdot (K + |N|)} = 0. \end{aligned}$$

While Eq. 3 is solvable using Cardano’s formulas, we are here only interested in the asymptotic behaviour of the optimal value of $\gamma$ in terms of varying values of *K*. Simplifying the solution of the equation shows, after some algebraic manipulation, that the optimal value of $\gamma$ is ${\mathcal {O}}(K^\frac{2}{3})$.

### Numerical evaluation of theoretical findings

As discussed in the previous section, the connection between the parameter of interest $\gamma$ and parameters *K*—maximum number of communities considered, *k*, the actual number of communities and |*N*|, and the number of nodes in a given network is given in Eq. 3. Being analytically intractable, we simulated the solutions of this equation numerically, over the following parameter space: values of *K*, ranging from 10 to 5,000 in the increments of 10, *k* ranging from 10 to 1,000 in the increments of 10 were considered. The number of nodes |*N*| were [500,1000,2500,5000,10000]. For each parameter combination, we solved the Eq. 3 with starting conditions [10,50,100] and averaged the results (for stability purposes). The results are finally fit to the estimated relation between *k* and $\gamma$, i.e.

$$\begin{aligned} \gamma = a \cdot \root 3 \of {K^{2}} + b. \end{aligned}$$

The agreement between the simulated results and the assumed relation between *K* and $\gamma$ is shown in Fig. 9 It can be observed that the agreement between the orange line (model) and the blue (simulated data) is a reasonably good fit for most of the *K* space.

## Appendix 2: Effects of embedding dimensionality on Silhouette

In this section we offer evidence which led us to introduce optional embedding-level normalization of Silhouette score, yielding more robust community detection. The non-normalized scores are shown in Fig. 10, whereas normalized ones are shown in Fig. 11. The plots show distributions of Silhouette scores across different embedding dimensions. Each distribution is based on grid search across the set of window sizes, as well as negative samples as discussed in Sect. 4.1.4.

The normalized Silhouette scores are computed as follows. Let $\text {Silhouette}(P(G))$ represent the Silhouette score obtained for a given *k* (number of clusters). Let $s_m$ represent the maximum Silhouette as identified using the implemented optimization and $s_i$ the *i*-th Silhouette considered when $k_i$ clusters were searched for. The normalized $s_m$, denoted ${\mathfrak {s}}_{m}$ is computed as:

$$\begin{aligned} {\mathfrak {s}}_{m} = \frac{s_m - \min {(s_i)}}{\max {(s_i)} - \min {(s_i})} \end{aligned}$$

Further, we observed that updating the best-performing embedding space based on mean normalized Silhouette values also yields more robust performance, yet evaluation of such claims is left for further work.

## Footnotes

1 If the binary vector was composed exclusively of ones, the iteration would compute the global PageRank vector, and the considered equation would be to reduce to the standard PageRank iteration.

2 https://docs.python.org/2/library/multiprocessing.html
